# First observation of electronic trap levels in freestanding GaN crystals extracted from Si substrates by hydride vapour phase epitaxy

**DOI:** 10.1038/s41598-019-43583-y

**Published:** 2019-05-09

**Authors:** Moonsang Lee, Chang Wan Ahn, Thi Kim Oanh Vu, Hyun Uk Lee, Eun Kyu Kim, Sungsoo Park

**Affiliations:** 1 Korea Basic Science Institute, Daejeon, 34133 Republic of Korea; 2 Department of Physics, Quantum-Function Research Laboratory, Hanyang University, Seoul, 133-791 Republic of Korea; 3 Advanced Nano-surface Research Group, Korea Basic Science Institute, Daejeon, 34133 Republic of Korea; 4 Department of Science Education, Jeonju University, Jeonju, 303 Republic of Korea; 5 Analytical Laboratory of Advanced Ferroelectric Crystals, Jeonju University, Jeonju, 303 Republic of Korea

**Keywords:** Electrical and electronic engineering, Inorganic LEDs, Characterization and analytical techniques, Inorganic LEDs, Spectrophotometry

## Abstract

The electronic deep level states of defects embedded in freestanding GaN crystals exfoliated from Si substrates by hydride vapour phase epitaxy (HVPE) is investigated for the first time, using deep level transient spectroscopy (DLTS). The electron traps are positioned 0.24 eV (E1) and 1.06 eV (E2) below the conduction band edge, respectively. The capture cross sections of E1 and E2 are evaluated to be 1.65 × 10^−17^ cm^2^ and 1.76 × 10^−14^ cm^2^ and the corresponding trap densities are 1.07 × 10^14^ cm^−3^ and 2.19 × 10^15^ cm^−3^, respectively. The DLTS signal and concentration of the electronic deep levels are independent of the filling pulse width, and the depth toward the bottom of the sample, evidenced by the fact that they are correlated to noninteracting point defects. Furthermore, Photoluminescence (PL) measurement shows green luminescence, suggesting that unidentified point defects or complex, which affect the optical characterisitics, exhibit. Despite the Si-based materials, the freestanding GaN exhibits deep level characteristics comparable to those of conventional freestanding GaN, suggesting that it is a desirable material for use in the next generation optoelectronic devices with the large-scalibilityand low production costs.

## Introduction

Owing to their unique physical properties, gallium nitride (GaN) and its related compounds are among the most promising materials for optoelectrical devices, such as laser diodes (LDs), ultra violet detectors, light-emitting diodes (LEDs), and high frequency and high power electronics^1,2^. To improve the performances of GaN-based devices, it is necessary to use freestanding (FS) GaN substrates. Because native bulk GaN does not exist, various growth methods, such as ammonothermal, high temperature high pressure (HTHP) growth, Na flux, and hydride vapour phase epitaxy (HVPE) have been employed to obtain FS-GaN to date^3–6^. Among these things, HVPE has good potential because it can provide a high growth rate and relatively high crystallinity^7^. Issues such as size limitations (<6 inch diameter) and high production costs, however, have to be overcome for the commercialization of HVPE FS-GaN wafers^8^. Although the introduction of Si substrates as a supporting material could help resolve such issues, this process has been considered impossible owing to the generation of cracks in HVPE GaN layers and meltback effects^9,10^. Researchers have tried to obtain FS-GaN crystals from Si substrates using diverse approaches, such as an intermediate buffer layer, graded buffer layers, and SiC nano buffer layers^11–13^. However, there have not been any reports on the growth of FS-GaN crystals extracted from Si substrates. Recently, we demonstrated the growth of crack-free FS-GaN 2 inch in diameter and 400 µm in thickness using *in situ* removal of the Si substrate by HVPE as well as its application in InGaN/GaN blue light emitting diodes (LEDs)^14,15^. However, the electrical properties of the defect states embedded in HVPE FS-GaN crystals detached from Si substrates have heretofore not been investigated. To appropriately utilize FS-GaN based on Si substrates, it is essential to explore and understand the origins and properties of defects in GaN layers peeled from Si substrates.

In this paper, we report the origin and electrical properties of electronic deep trap levels incorporated in the HVPE FS-GaN crystals extracted from Si substrates, using deep-level transient spectroscopy (DLTS). We expect that this study will shed light on the characterization of electronic states embedded in FS-GaN crystals peeled from Si substrates by HVPE, thus providing impetus to the performance optimization of optoelectronic devices that use FS-GaN crystals based on Si substrates.

## Experimental

**Figure 1:**
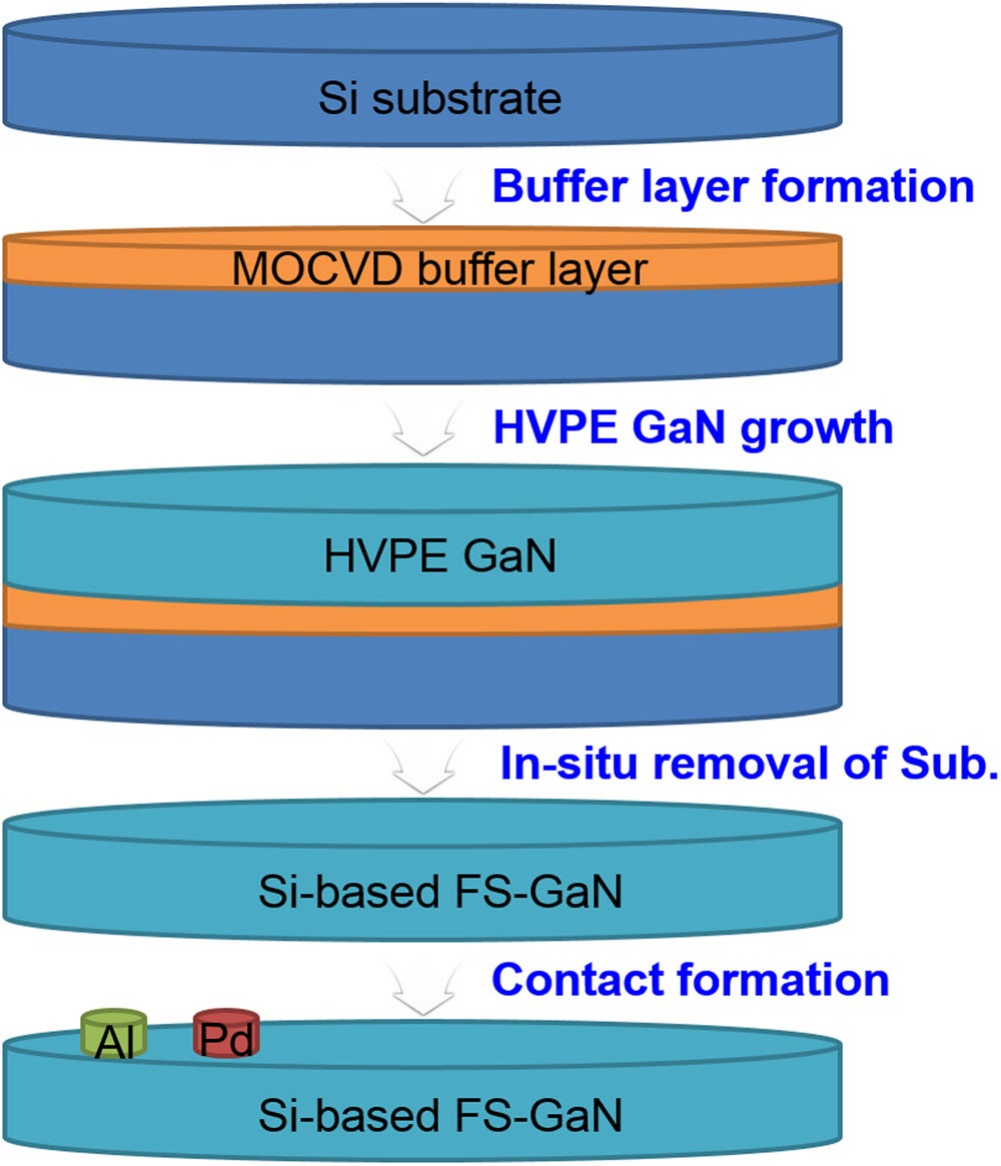
Schematic illustration for the formation of Si-based FS-GaN Schottky diodes.

The FS-GaN crystals used in this study were grown from Si substrates, using an *in-situ* removal method by HVPE. The details of the growth process are described elsewhere^15,16^. A home-built vertical type HVPE system with an upstream HCl gas channel was used to achieve the FS-GaN crystal from a Si substrate. To form Si-based HVPE GaN layers, AlN/Al_0.4_Ga_0.6_N buffer layers were deposited on 2 inch Si (111) substrate, using metalorganic chemical vapour deposition (MOCVD). Next, HVPE GaN films were grown in atmosphere pressure with V/III ratio of 20, followed by the *in-situ* etching of the substrate at 1273 K. Finally, we could obtain the Si-based FS-GaN. To investigate the electrical characterization of the FS-GaN crystals, Schottky diodes were formed. First, 3-mm-diameter Al metal (150 nm) was deposited on one Ga-face of the FS-GaN to form Ohmic contacts, using an electron beam evaporator. Subsequently, 300-µm diameter Pd Schottky contacts were formed on the other Ga-face surface by the electron beam evaporator, followed by rapid thermal annealing in ambient Ar at 823 K to improve the contact formation. The overall process to form S-based FS-GaN Schottky diodes is illustrated in Fig. 1. The measurements of deep levels in the FS-GaN were performed in a system developed in-house, using a 100 mV signal at 1 MHz within a temperature range of 100–420 K. Moreover, the optical characteristics of the FS-GaN crystals were measured by photoluminescence (PL) analysis, which was excited using a He–Cd laser of 325 nm wavelength at room temperature.

## Results and Discussion

**Figure 2:**
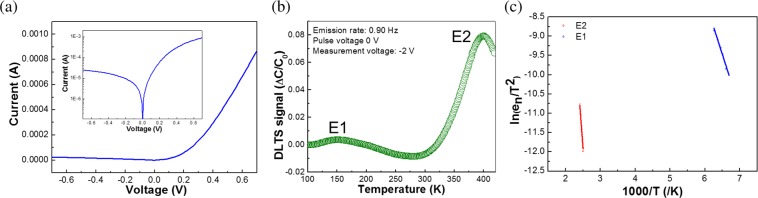
(**a**) *I*–*V* characteristics of Pd/Si-based FS-GaN Schottky diode at room temperature. The inset depicts logarithmic plot of *I*–*V* characteristics in Pd metal/Si-based Schottky diode. (**b**) DLTS spectrum of Si-based FS-GaN measured under a pulse voltage of 0 V and an applied voltage of −2 V in the temperature range of 100‒420 K. (**c**) Arrhenius plot of the DLTS signal of the studied material.

A typical current-voltage (*I*–*V*) measurements of Pd/Si-based FS-GaN Schottky diode were employed at room temperature, as shown in Fig. 2(a). The diode exhibits a clear rectifying characteristics, implying that the Schottky junctions are well formed at metal/Si-based FS-GaN. Furthermore, the inset of Fig. 1(a) obviously represents that the forward and reverse leakage current increase exponentially with the bias, accompanying the shape of a thermionic field emission and trap-assisted tunneling^17^. This indicates that deep traps are present in the Si-based FS-GaN^18^. The detailed analysis of the *I*–*V* curves will be elsewhere. To investigate the deep trap states incorporated in the Si-based FS-GaN crystals, DLTS measurements and their Arrhenius plots were conducted, respectively, as illustrated in Fig. 2(b,c). The DLTS spectrum was obtained at an emission rate (e_n_) of 0.90 Hz under an applied filling pulse width of 20 ms, within a temperature range of 100‒420 K. Two distinct deep level states, which are labelled as E1 and E2, can be seen.

The electronic states of E1 and E2 were centered at 0.24 eV and 1.06 eV below the conduction band edge, respectively. It is apparent that the trap E2 behaves as the dominant defect with a trap density of 2.19 × 10^15^/cm^3^ and a capture cross section of 1.76 × 10^−14^/cm^2^. A trap density and a capture cross section of E1 were estimated to be 1.07 × 10^14^/cm^3^ and 1.65 × 10^−17^/cm^2^, respectively, as shown in Table 1. Compared to the properties of deep levels in conventional HVPE FS-GaN crystals, the fingerprints of electronic states, which indicate a trap density and capture cross section, embedded in the Si-based FS-GaN are comparable^19–22^. (See Table 2) Furthermore, this is also comparable to the characteristics of the deep levels in the GaN layers grown using metalorganic chemical vapour deposition (MOCVD)^23–25^. Notwithstanding HVPE with a high growth rate, which can cause a deterioration of crystal quality in the GaN layers, the properties of deep trap levels in the Si-based HVPE FS-GaN exhibit relatively nondegraded characteristics, compared to those of the preexisting GaN films. This strongly suggests that the Si-based FS-GaN crystals can realize large-scale high-performance opto-electronic devices without any electronic degradation.

**Table 1:** Defect parameters for the FS-GaN crystal extracted from a Si substrates by HVPE.

Defect	Activation energy (eV)	Capture cross section (cm^2^)	Trap density (cm^−3^)
E1	0.24	1.65 × 10^−17^	1.07 × 10^14^
E2	1.06	1.76 × 10^−14^	2.19 × 10^15^

**Table 2:** Summary of defect parameters in Si-based FS-GaN compared to other literatures.

Ref.	Activation energy (eV)	Capture cross section (cm^2^)	Trap density	Emission rate (Hz)
This study	0.24 1.06	1.65 × 10^−17^ 1.76 × 10^−14^	1.07 × 10^14^ 2.19 × 10^15^	0.90
^19^	0.25, 0.563, 0.65, 0.69, 1.40, 1.55	10^−12^‒10^−16^	~10^12^‒2.2 × 10^15^	0.014
^20^	0.25, 0.35, 0.59, 0.66, 1.0	6.7 × 10^−14^‒9.0 × 10^−16^	Mid-10^14^	0.0125‒1.25
^21^	0.25, 0.35, 0.53, 0.58, 0.70	1.2 × 10^−15^‒2.4 × 10^−15^	—	—
^22^	0.25, 0.6, 0.85, 1.0	—	≤10^14^	—

**Figure 3:**
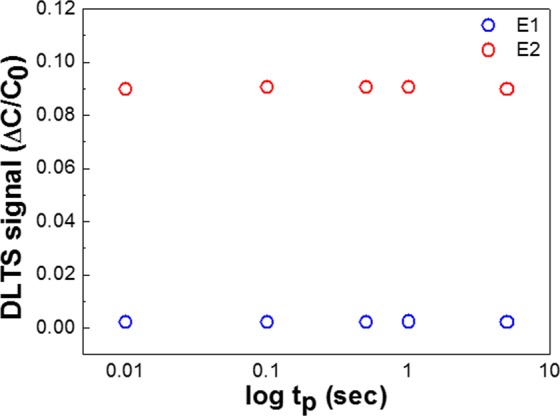
DLTS signals of E1 and E2 as a function of filling pulse time t_p_.

To shed light on the origin of the trap states in the Si-based FS-GaN, we measured the deviation of the DLTS signals as a function of filling pulse time (t_p_), as depicted in Fig. 3. It is clear that the variations of the signals for the two deep levels are negligible. This implies that the two electronic deep levels originate from noninteracting defects, namely point ones. It is well known that the noninteracting defects depend exponentially on t_p_. On the contrary, interacting defects related to dislocations and stacking faults, are proportional to ln(tp). Because the outlines of a DLTS signal as a function of ln(tp) in Fig. 2 are independent of ln(t_p_), we can define the two defects as noninteracting point defects. As mentioned above, the E1 and E2 traps have been commonly observed in other GaN layers. E1 was considered as nitrogen or nitrogen vacancy (V_N_)-related defects, denoted as V_Ga_-V_N_ pair^24,26,27^, and E2 is interpreted as nitrogen interstitial (N_I_)^28^ or line defects^29,30^.

**Figure 4:**
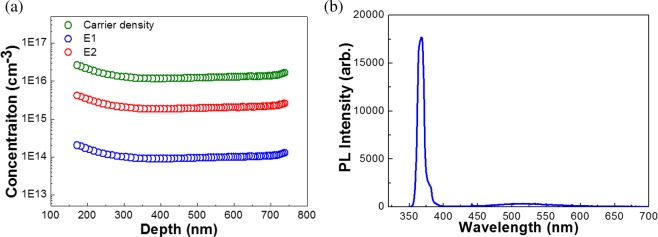
(**a**) Concentrations of carrier density and trap densities of E1 and E2 as a function of the distance from the surface of the Si-based FS-GaN. (**b**) Room temperature PL spectrum of the Si-based FS-GaN.

Figure 4 illustrates the depth profiles of the trap densities of E1 and E2 and carrier concentration in the Si-based FS-GaN, obtained from the DLTS signal and capacitance-voltage profile, respectively. The depth from the surface of the Si-based FS-GaN became intense with increasing reverse bias voltage. As shown in Fig. 4(a), the behaviors of the two defect densities are similar, and these were almost unchanged throughout the sample depth. Slight increases in the lines at a depth of 200 nm were found. Surface defects due to electron-beam evaporation are responsible for such a feature^31^. Considering the nonlinear relationship between the DLTS signal and ln (t_p_) and concentration depth profiles of E1 and E2, we concluded that these defects is inherited from the noninteracting point defects without any extended defect-related ones. Additionally, we implemented PL analysis of the studied materials, as presented in Fig. 4(b). Strong band-edge emission peaks of the Si-based FS-GaN crystals appeared near 3.375 eV^32^. The full width at half maximum (FWHM) of the edge peak was approximately 8.5 nm. The PL peaks of the samples exhibited a red-shift of 96 meV with respect to those of completely relaxed bulk GaN (3.471 eV), indicating the presence of tensile stress in the FS-GaN^33,34^. This is attributed to the crystal quality difference between the Ga- and N-face surfaces of the FS-GaN^35^. It is essential to note that the slight plateau around wavelength of 514.5 nm can be clearly observed. Indeed, the typical yellow bands are positioned at 2.1‒2.3 eV. However, broad band with maxima at 514.5 nm in the Fig. 4 can simulate green band rather than yellow luminescence. Even if a number of the exploration for the green emission from the GaN materials were implemented, its origin has not been clearly discovered up to now^36,37^. Indeed, some literatures stated that it may be associated with V_N_, and V_Ga_^38^. Based on PL and DLTS analyses, the Si-based FS-GaN may involve high nitrogen concentration. Note that the two defects are related to V_Ga_, V_N_, V_Ga_-V_N_ pair, N_I_-related defects, indicating high nitrogen fraction in the GaN films. Lymperakis *et al*. also stressed that the high strain field of the dislocation in the GaN layers can induce a metal-like structure of the Ga–Ga bonds^39^. Moreover, they addressed that these strain-induced metallic bond states can be observed in the GaN crystals grown by MOCVD or HVPE, which contain the abundant stress and nitrogen concentration in the layer^39^. It is noticeable that Si-based FS-GaN underwent a huge tensile strain during the HVPE growth^16^. Furthermore, we also observed metallic Ga-Ga bonding in GaN template on a Si substrate, which underwent high tensile strain during the growth stage^40^. However, we cannot observe any evidence on the electronic deep level states related to the metallic Ga bonding in Si-based FS-GaN. Consequently, the characteristics of the electronic states of FS-GaN crystal extracted from a Si substrate is comparable to that of conventional freestanding GaN layers, despite of using a Si material. This indicates that Si-based FS-GaN can be a promising material to achieve the high performance opto-electronic devices with large scalability and low cost. To verify the energy states of the metallic bonds, electron energy loss spectroscopy (EELS) combined with scanning transmission electron microscopy (STEM) analysis is under investigation.

## Conclusion

The electronic states of the deep levels in Si-based FS-GaN were investigated using DLTS analysis. Two deep trap levels were observed at E_c_ − 0.24 eV and E_c_ − 1.06 eV with capture cross sections of 1.65 × 10^−17^ cm^−2^ and 1.76 × 10^−14^ cm^−2^ and trap densities of 1.07 × 10^14^ cm^3^ and 1.19 × 1015 cm^3^, respectively. The DLTS signals as a function of filling pulse time and trap density depth profiles prove that the two deep levels are involved in noninteracting point defects. The PL measurement revealed that the green emission was observed, inherited from unknown sources. Despite of originating from a Si substrate, The properties of the electronic deep level states of Si-based FS-GaN is comparable to those of conventional one. We believe that the Si-based FS-GaN exhibits the desirable characteristics for large-scale opto-electronic applications with low production costs.

## Methods

The fingerprints of electronic deep centers including the activation energy (E_a_) and capture cross section (σ_n_) of trap states, extracted from Arrhenius plots, can be evaluated as follows^41^:

1 $${\rm{In}}(\frac{{{\rm{e}}}_{{\rm{n}}}}{{{\rm{T}}}^{2}})={\rm{In}}(\sqrt{6}{\rm{\pi }}1.5{k}^{2}{{\rm{m}}}_{{\rm{n}}}^{\ast }{{\rm{\sigma }}}_{{\rm{n}}}/{{\rm{h}}}^{3})+(\,-\,{{\rm{\Delta }}E}_{{\rm{a}}}/1000{\rm{k}})\,1000/{\rm{T}}$$

where e_n_, T, k, m*, and h indicate the emission rate, the absolute temperature, Boltzmann’s constant, the effective mass of the carrier, and Plank’s constant, respectively. The trap parameters are presented in Table 1.

Furthermore, the trap density can be determined as a following equation^42^:

2 $${{\rm{N}}}_{{\rm{t}}}=2{{\rm{N}}}_{{\rm{d}}}{{\rm{F}}}^{-1}{\rm{\Delta }}C/{\rm{C}}$$

where N_d_, ∆C, and F indicate the donor concentration, the capacitance change during relaxation, and the spectrometer function of 3.5 used in this work, respectively.
